# TEP SNORD12B, SNORA63, and SNORD14E as novel biomarkers for hepatitis B virus-related hepatocellular carcinoma (HBV-related HCC)

**DOI:** 10.1186/s12935-023-03179-z

**Published:** 2024-01-02

**Authors:** Xuan Zhao, Guanxuan Chen, Yawen Wu, Xiao Li, Zhe Zhang, Li Xie, Xianrang Song, Xingguo Song

**Affiliations:** 1 Department of Clinical Laboratory, Shandong Cancer Hospital and Institute, Shandong First Medical University, Shandong Academy of Medical Sciences, Jinan, Shandong PR China; 2 Department of Intensive Care Unit, Shandong Cancer Hospital and Institute, Shandong First Medical University, Shandong Academy of Medical Sciences, Jinan, Shandong PR China; 3 Shandong Provincial Key Laboratory of Radiation Oncology, Shandong Cancer Hospital and Institute, Shandong First Medical University, Shandong Academy of Medical Sciences, Jinan, Shandong PR China

**Keywords:** Tumor-educated platelets (TEPs), HBV-related HCC, snoRNAs, SNORD12B, SNORA63, SNORD14E, Biomarker

## Abstract

**Purpose:**

The alterations of RNA profile in tumor-educated platelets (TEPs) have been described as a novel biosource for cancer diagnostics. This study aimed to explore the potential snoRNAs in TEP as biomarkers for diagnostics of hepatitis B virus-related hepatocellular carcinoma (HBV-related HCC).

**Methods:**

Platelets were isolated using low-speed centrifugation and subjected to a quantitative polymerase chain reaction (qPCR) for snoRNAs detection.

**Results:**

Down-regulated SNORD12B and SNORD14E as well as up-regulated SNORA63 were identified in TEP from HBV-related HCC, which could act as diagnostic biomarkers for HBV-related HCC as well as the early disease. Besides, TEP SNORD12B, SNORD14E, and SNORA63 facilitate the diagnostic performance of AFP and achieve favorable diagnostics efficiency for HBV-related HCC when combined with platelet parameters.

**Conclusions:**

Aberrant expression of SNORD12B, SNORA63, and SNORD14E in TEPs could serve as the novel and non-invasive biomarkers for HBV-related HCC diagnosis.

**Supplementary Information:**

The online version contains supplementary material available at 10.1186/s12935-023-03179-z.

## Introduction

Hepatocellular carcinoma (HCC), the most common pathological type of primary liver cancer, accounts for over 80% of all cases with rapidly increasing incidence and mortality in recent decades with complicated pathogenesis and molecular biology [1–3]. China has one of the highest hepatitis B virus (HBV)-carrier prevalences in the world and is also estimated to have the highest incidence of HCC. HBV infection has been identified as one of the main causes and driving forces in HCC tumorigenesis and development, so-called HBV-related HCC [4], contributing to the largest population of HCC [5]. A previous study estimated that HBV contributed to about 59.3% of liver cancer in China [6], Unfortunately, due to a lack of ideal diagnostic biomarkers with favorable sensitivity and specificity, most patients have advanced disease at the time of first diagnosis, resulting in poor prognosis and high mortality of HBV-related HCC.

Platelets, as one of the principal constituents of the blood, are versatile non-nuclear cell fragments originating from bone marrow megakaryocytes, serving much more comprehensive functions in all steps of tumorigenesis, including tumor development and metastasis [7], so-called tumor-educated platelets (TEPs). TEPs exploit functional spliceosomes, a complex of small ribonucleoproteins (snRNPs) composed of splicing proteins and s small nuclear RNAs (snRNAs), empowering them to undergo splice events in response to signals released by cancer cells and the tumor microenvironment, in turn directly splice the circulating mRNA providing TEPs with a highly dynamic mRNA repertoire, with potential applicability to cancer diagnostics [8, 9]. Interestingly, small nucleolar RNAs (snoRNAs) have also been described as detectable in anucleate platelets. They participate in alternative splicing of pre-mRNA in platelets other than regulation of translation in nucleated cells [10].

SnoRNAs are a specific class of small, abundant, and stable non-coding endogenous RNAs with a length of 60 to 200 nucleotides that localize in the nucleolus, guiding the 2′-O-ribose methylation and psuedouridylation of rRNAs or snRNAs [11, 12]. Accumulating evidence has revealed that snoRNAs play a crucial role in tumorigenesis and progression, and serve as the non-invasive biomarker for cancer diagnostics. For example, SNORD88C, up-regulated in tissue and plasma of non-small cell lung cancer (NSCLC), served as the circulating biomarker and guided 2’-O-methylation of 28 S rRNA then to regulate SCD1 translation and promote growth and metastasis [13]. Aberrant expression and diagnostic potential of snoRNAs have been also observed in TEP. For example, our previous study demonstrated that TEP SNORD55 was significantly decreased in NSCLC patients [14], acting as a promising diagnostic biomarker.

In the present study, we aimed to investigate the diagnostic role of TEP snoRNAs in HBV-related HCC. We screened TEP SNORD12B, SNORA63, and SNORD14E based on a database and validated diagnostic efficacy in a large cohort followed by an initial exploration of their expression patterns, thus providing evidence for TEP snoRNAs as an efficient biomarker for HBV-related HCC.

## Materials and methods

### Database

The clinical information of HCC and paired adjacent tissue samples were provided by TCGA (http://cancergenome.nih.gov) and SNORic (http://bioinfo.life.hust.edu.cn/SNORic) database to analyze the expression of snoRNAs for HCC with 372 tumors and 50 adjacent normal tissues.

### Patients and clinical samples

117 treatment naïve patients with HBV-related HCC and 117 healthy volunteers admitted to Shandong Cancer Hospital and Institute from October to December 2022 were enrolled in the current study. The tumor stage was determined based on the 8th edition of the TNM staging criteria for liver cancer. The healthy donors showed no disease.

### Platelet isolation and RNA extraction

A low-speed centrifugation method was applied to isolate the platelet [6]. Plasma was collected in EDTAK2-coated purple-cap vacutainer tubes (BD) and then centrifuged twice for 10 min at 120 × g to collect the platelet-rich plasma (PRP), which was then subjected to 360 × g concentration for 20 min at room temperature to pellet the platelets. For RNA isolation of the platelets, Trizol reagent (Thermo Fisher Scientific, Carlsbad, CA, USA) was used to extract total RNA according to the introduction.

### Liu staining

Fresh PRP was spread on a microscope slide, then treated with Liu A and B solutions, and observed under an optical microscope.

### Reverse transcription and quantification by real-time PCR

Reverse transcription was performed using the miRNA First Strand cDNA Synthesis Kit (AG11716). Subsequently, qRT-PCR was performed on LC480 (Roche Diagnostics, Germany) using SYBR Green Premix Pro Taq HS qPCR Kit (AG11701). The relative expression of snoRNA was evaluated using Ct^snoRNA^ -Ct^U6^ [15]. The qRT-PCR primers are listed in Table 1. Each sample was provided with a secondary well, which was amplified in two copies at the same time to obtain the average cycle threshold (Ct) to reduce errors.

**Table 1:** Primer sequences involved

**SNORD12B -F** TTTTTCCCCGACAGATCGAC
**SNORD12B -R** GCTCAAGCTGGCATATCAGAC
**SNORD14E-F** TGGTCCAAAACATTCGCGGT
**SNORD14E-R** CATCCAAGGAAGGTAGTTGCCA
**SNORA63-F** TGCTAAGTGCTGTGTTGTCGTTCC
**SNORA63-R** TCCCTGGCTGCTACAGGAGAATAG
**U6-F** TGGAACGCTTCACGAATTTGCG
**U6-R** GGAACGATACAGAGAAGATTAGC

### Statistical analysis

SPSS 26.0 (IBM Corp., NY, USA) and GraphPad Prism 9.0 (GraphPad, CA, USA) were utilized to analyze the data. The Kolmogorov-Smirnov test was used to test the normality of the data. If normal, the unpaired t-test was used for the analysis of two groups of data and a one-way analysis of variance (ANOVA) for the analysis of multiple groups. If not, the Mann-Whitney U test was used for two groups of data and the Kruskal-Wallis test for multiple groups of analysis. Diagnostic efficiency was evaluated using receiver operating characteristic curves (ROC). The results were represented as mean ± SD, *p*-values < 0.05 was considered statistically significant.

## Results

### Aberrant expression of TEP SNORD12B, SNORA63, and SNORD14E in HBV related-HCC

To screen out the differential expression levels in liver hepatocellular carcinoma (LIHC) 372 tumor tissues and 50 control tissues along with their clinical characteristics from the TCGA and SNORic database were downloaded and included in the analysis. Differential expression of snoRNAs between between HCC and neighboring tissues was identified according to the the following criteria: adjusted *p* value < 0.05 and an absolute log2 (FC) > 0.5 or <-0.5. Then, the peer selected snoRNAs were detected in the plasma from a small-size cohort including 24 HCC patients and 24 healthy volunteers. Unexpectedly, most were excluded due to low expression levels as well as no difference in expression in plasma unlike those in tissues, SNORD12B, SNORA63, and SNORD14E were finally considered as the candidates.

**Fig. 1:**
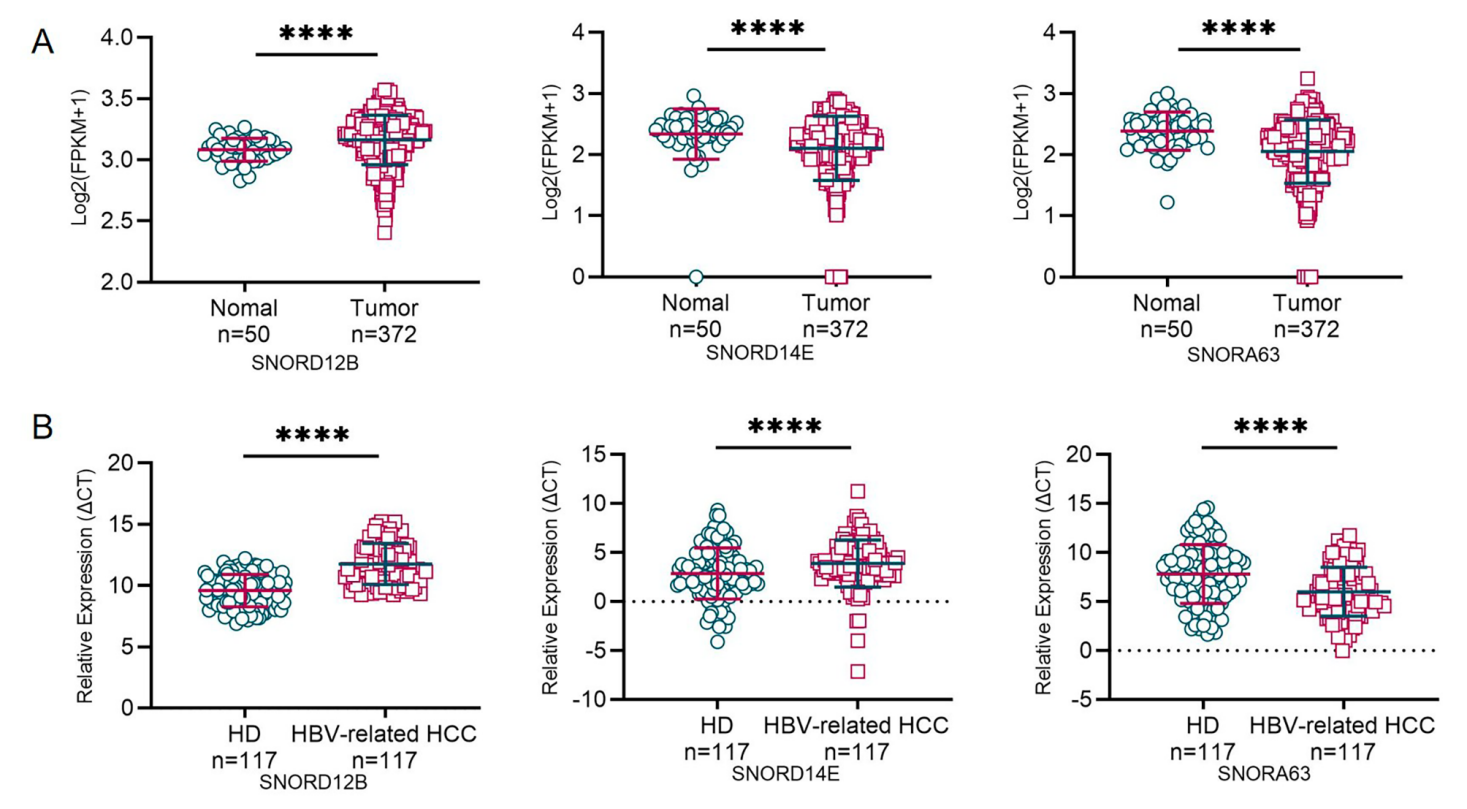
Aberrant expression of TEP SNORD12B, SNORA63, and SNORD14E in HBV-related HCC. **(A)** The SNORic database was used to analyze the differential expression of SNORD12B, SNORA63, and SNORD14E in cancer and adjacent tissues. **(B)** Differential expression of platelet SNORD12B, SNORA63, and SNORD14E in 117 healthy individuals and 117 patients with HBV-related HCC. The expression level of snoRNAs in the database is represented by log2 (FPKM + 1). (HD: healthy donors; FPKM: fragments per kilobase of exon model per million mapped fragments; ****p < 0.0001)

We first confirmed their differential expressions in the public database. As shown in Fig. 1A, upregulated SNORA63 and SNORD14E, as well as downregulated SNORD12B were observed in HCC tissues compared to the control. Then, we verified their expression in TEPs from 117 HBV-related HCC patients and 117 healthy donors. SNORA63 was higher along with SNORD12B was lower in the TEPs from HBV-related HCC than those from the healthy, consistent with their expression in HCC tissue; Nevertheless, TEP SNOD14E differed from tissue expression (Fig. 1B). We also analyzed the correlation between snoRNA expression and clinical characteristics. As listed in Table 2, SNORD14E was found to be closely associated with the N stage, while no other significant correlations were observed.

**Table 2:** SNORNAs expression levels of HCC in relation to clinical and pathological

Parameters in a series of 117 HCC	Samples	SNORD12B		SNORD14E		SNORA63	
		Mean ± SD	*P* value	Mean ± SD	*P* value	Mean ± SD	*P* value
**Male**	92	11.81 ± 1.642	0.5573	3.942 ± 2.397	0.5266	6.048 ± 2.51	0.8186
**Female**	25	11.63 ± 1.818		3.706 ± 2.485		5.918 ± 2.502	
**Age(Year ≤ 60)**	57	11.78 ± 1.468	0.6668	3.891 ± 2.5	0.8375	5.71 ± 2.396	0.1914
**Age(Year>60)**	60	11.77 ± 1.863		3.893 ± 2.336		6.315 ± 2.575	
**Not-smoking**	72	11.8 ± 1.769	0.99	3.677 ± 2.655	0.8638	5.947 ± 2.48	0.6884
**Smoking**	45	11.73 ± 1.531		4.236 ± 1.924		6.138 ± 2.55	
**Not-drinking**	73	11.85 ± 1.825	0.7654	3.6 ± 2.584	0.3747	5.905 ± 2.533	0.5209
**Drinking**	44	11.65 ± 1.403		4.375 ± 2.016		6.212 ± 2.455	
**TNM Stage**							
**I**	17	11.46 ± 1.721	0.2227	4.415 ± 2.164	0.1516	5.483 ± 1.854	0.5497
**II**	37	12.28 ± 1.874		4.148 ± 2.746		6.18 ± 2.532	
**III**	45	11.63 ± 1.445		3.636 ± 2.254		6.23 ± 2.494	
**IV**	18	11.4 ± 1.626		3.51 ± 2.293		5.676 ± 3.011	
**T Stage**							
**T1**	22	11.23 ± 1.651	0.1148	3.68 ± 3.057	0.1383	4.885 ± 2.094	0.1029
**T2**	9	11.1 ± 1.599		2.717 ± 1.029		5.737 ± 2.686	
**T3**	34	11.56 ± 1.446		3.645 ± 2.115		5.99 ± 2.59	
**T4**	25	12.2 ± 1.672		4.163 ± 2.595		6.727 ± 2.665	
**Not available**	27	12.32 ± 1.829		4.515 ± 2.239		6.423 ± 2.288	
**LN metastasis**							
**N1**	72	11.62 ± 1.614	0.2762	3.755 ± 2.483	**0.043**	5.927 ± 2.462	0.271
**N2**	14	11.08 ± 1.39		3.32 ± 2.551		5.116 ± 2.733	
**Not available**	31	12.45 ± 1.757		4.468 ± 2.108		6.645 ± 2.395	
**Distant metastasis**							
**M1**	74	11.62 ± 1.613	0.7592	3.706 ± 2.46	0.3919	5.952 ± 2.496	0.6624
**M2**	15	11.48 ± 1.608		3.687 ± 2.482		5.631 ± 2.995	
**Not available**	28	12.34 ± 1.798		4.492 ± 2.2		6.41 ± 2.246	

**Bold values indicate statistically significant*p*values** (***p* < 0.05)**

We also evaluated the snoRNA expression in non-HBV related-HCC. As shown in Fig. S1, downregulated SNORD12B and SNORD14E, as well as upregulated SNORA63 were observed in non-HBV related-HCC tissues compared to the control, consistent with the result in HBV related-HCC. Notably, SNORD12B was increased in non-HBV related-HCC compared that in HBV related-HCC, may be too little concerned with the number of cases in the non-HBV-related group.

### TEP SNORD12B, SNORA63, and SNORD14E as novel diagnostic biomarkers for HBV-related HCC

**Fig. 2:**
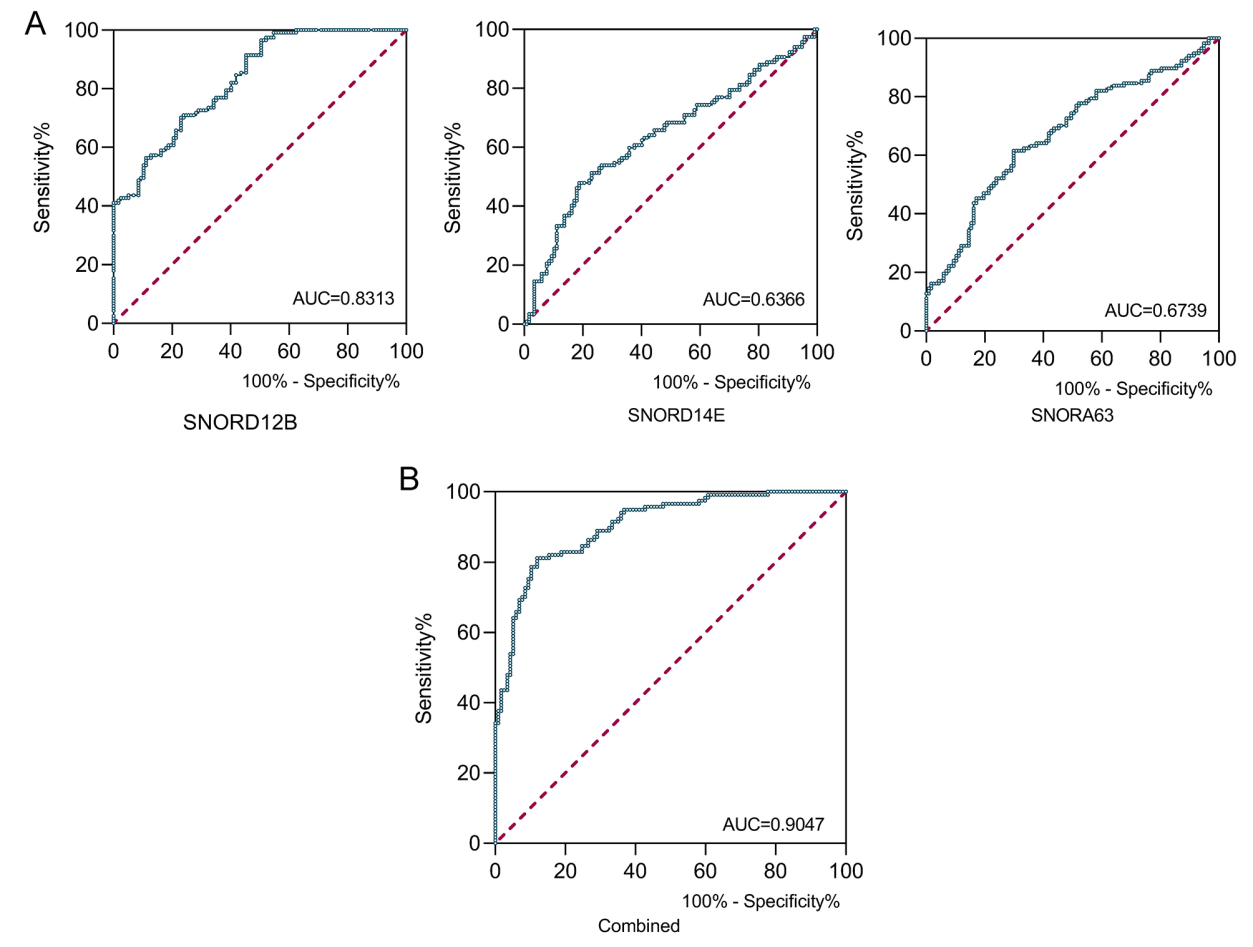
TEP SNORD12B, SNORA63, and SNORD14E as novel diagnostic biomarkers for HBV-related HCC. **(A)** ROC curve analysis of TEP SNORD12B (left, AUC = 0.8313), SNORA63 (mid, AUC = 0.6366), and SNORD14E (right, AUC = 0.6739) was performed for HBV-related HCC; **(B)** The diagnostic performance of the three combined TEP snoRNAs possessed AUC of 0.9047. (AUC: area under curve.)

Furthermore, a receiver-operating characteristic (ROC) curve was employed to evaluate the diagnostic accuracy of the above three snoRNAs in TEPs for HBV-related HCC. TEP SNORD12B, SNORA63, and SNORD14E showed AUCs (Area Under Curve) of 0.8313, 0.6366, and 0.6739, respectively (Fig. 2A). Notably, their combined diagnostic efficiency possessed an AUC of 0.9047 (Fig. 2B and Table S1). Taken together, our data suggest that TEP snoRNAs: SNORD12B, SNORA63, and SNORD14E possess the potential to the promising biomarkers for HBV-related HCC.

### TEP SNORD12B, SNORA63, and SNORD14E can be used as neoteric diagnostic biomarkers for early-stage HBV-related HCC

**Fig. 3:**
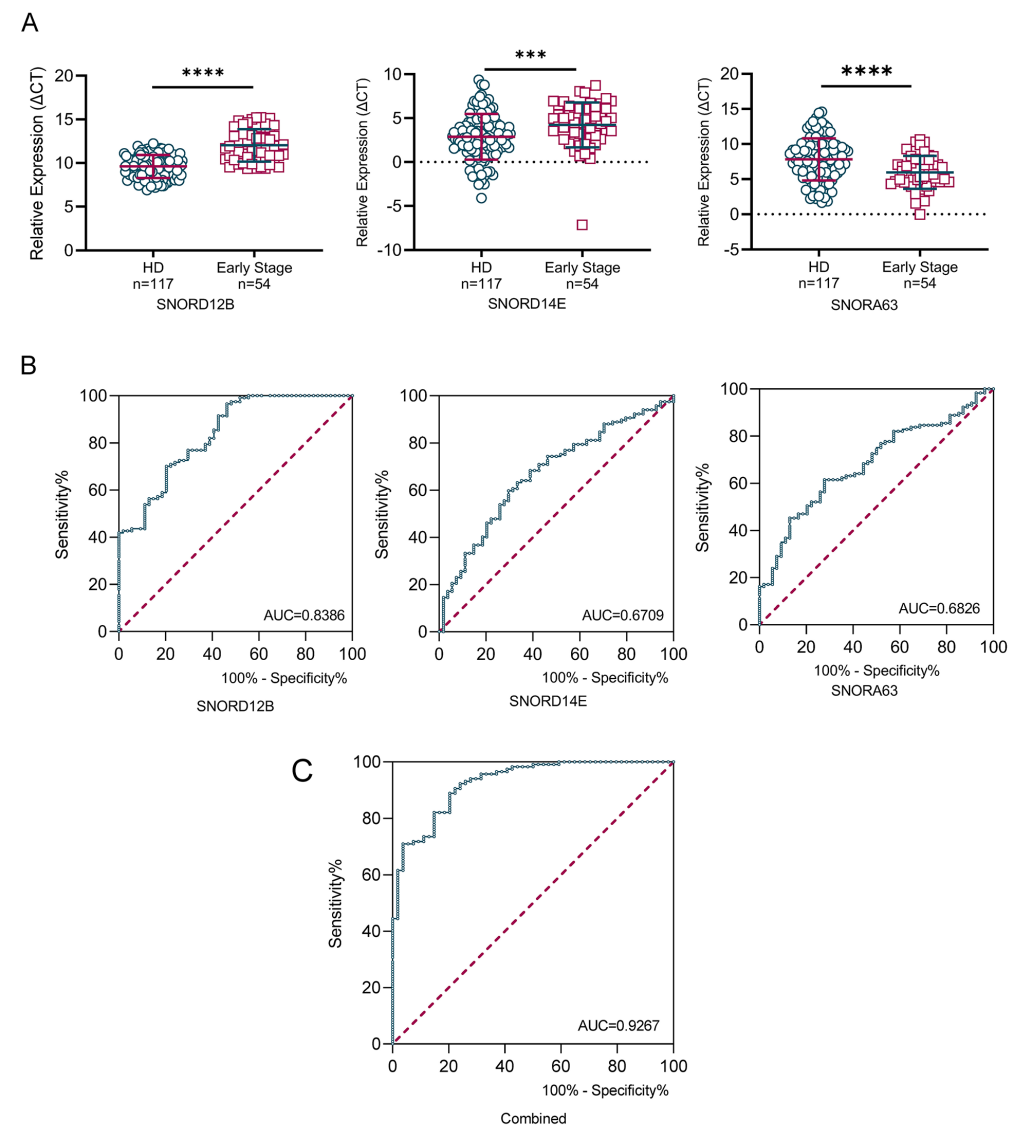
TEP SNORD12B, SNORA63, and SNORD14E can be used as neoteric diagnostic biomarkers for early-stage HBV-related HCC. **(A)** Differential expression of TEP SNORD12B, SNORD14E, and SNORA63 in healthy donors and patients with early-stage HBV-related HCC. **(B)** The AUCs of TEP SNORD12B, SNORA63, and SNORD14E in early HBV-associated HCC was 0.8386, 0.6709, and 0.6826, respectively. **(C)** The diagnostic performance of the three TEP snoRNAs combined showed an AUC of 0.9267. (AUC: area under curve. ***p < 0.001, ****p < 0.0001)

We also examined the differential expression of these three TEP snoRNAs between early-stage HBV-related HCC patients (I + II phage) and healthy individuals. As shown in Fig. 3A, TEP SNORD12B and SNORD14E levels were significantly lower, while TEP SNORA63 level was higher in early-stage patients compared to healthy individuals.

Similarly, ROC analysis of these three snoRNAs in TEP was also applied in the early-stage patients compared to healthy individuals. As shown in Fig. 3B and Table S2, TEP SNORD12B, SNORA63, and SNORD14E addressed the favorable diagnostic efficiency for early diagnostics, possessing the AUCs of 0.8386, 0.6709, 0.6826, respectively, as well as 0.9267 when their three were combined, indicating the promising diagnostic performance of TEP snoRNA.

### TEP SNORD12B, SNORD14E, and SNORA63 facilitate the diagnostic performance of AFP for HBV-related HCC

**Fig. 4:**
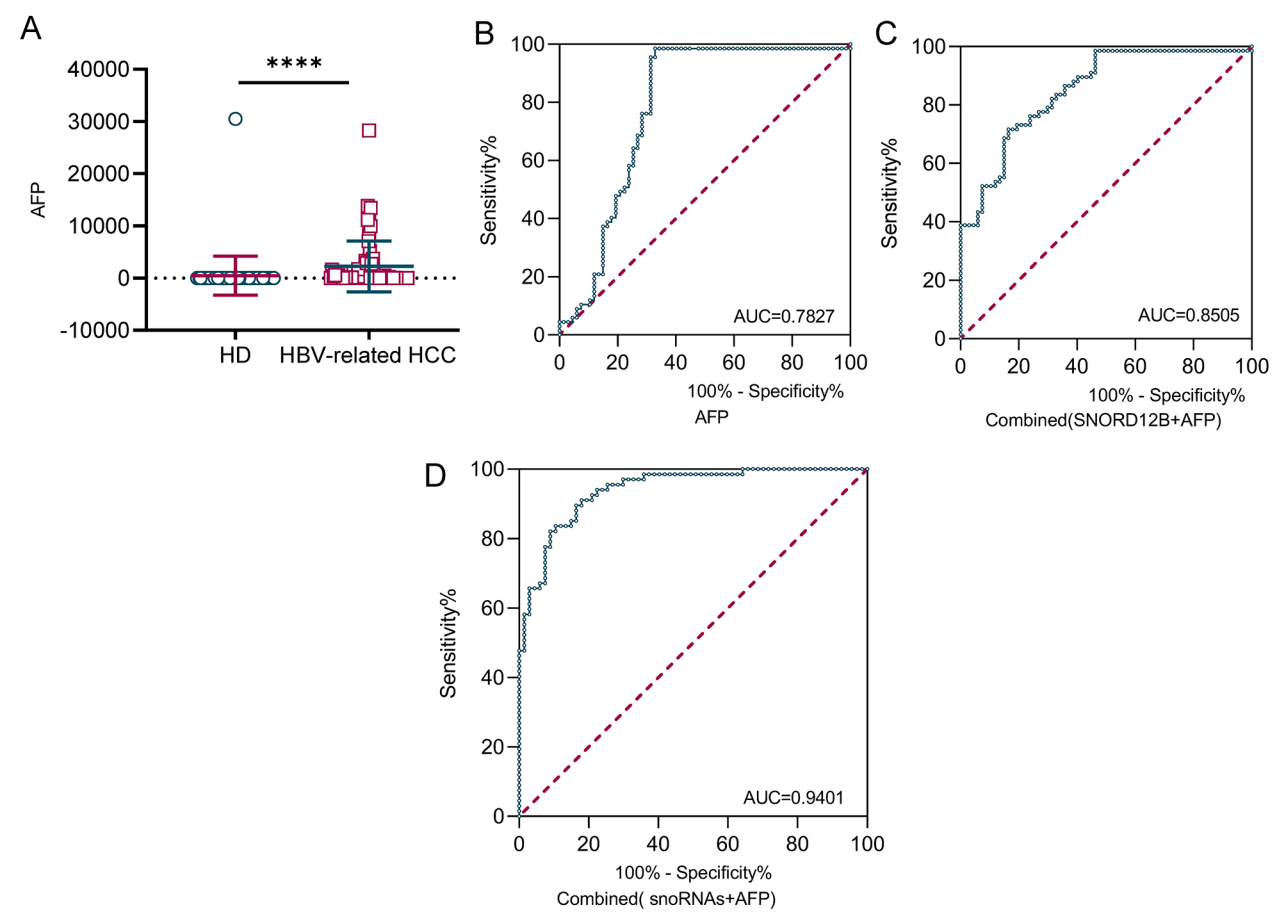
TEP SNORD12B, SNORD14E, and SNORA63 facilitate the diagnostic performance of AFP for HBV-related HCC. **(A)** Differential level of plasma AFP between healthy donors and HBV-related HCC patients. **(B-D)** TEP snoRNAs facilitate the AFP diagnostic performance. The ROC curve analysis of AFP for HBV-related HCC patients **(B)**, combined with SNORD12B **(C)**, and all three snoRNAs **(D)** (AUC: area under curve; ****p < 0.0001)

Alpha-fetoprotein (AFP) is the most common clinically used diagnostic and prognostic biomarker for HCC. We first detected AFP levels in healthy subjects and HBV-related HCC patients (Fig. 4A), then analyzed its diagnostic accuracy using the ROC curve (Fig. 4B), the AUC of AFP was 0.7872 with 98.51% sensitivity and 67.16% specificity. Notably, the AUC of AFP was elevated to 0.8505 when combined with TEP SNORD12B, possessing the best performance in the three TEP snoRNAs (Fig. 4C), and to 0.9401 when combined with these three snoRNAs (Fig. 4D and Table S3).

### TEP snoRNAs combined with platelet parameters achieve favorable diagnostics efficiency for HBV-related HCC

**Fig. 5:**
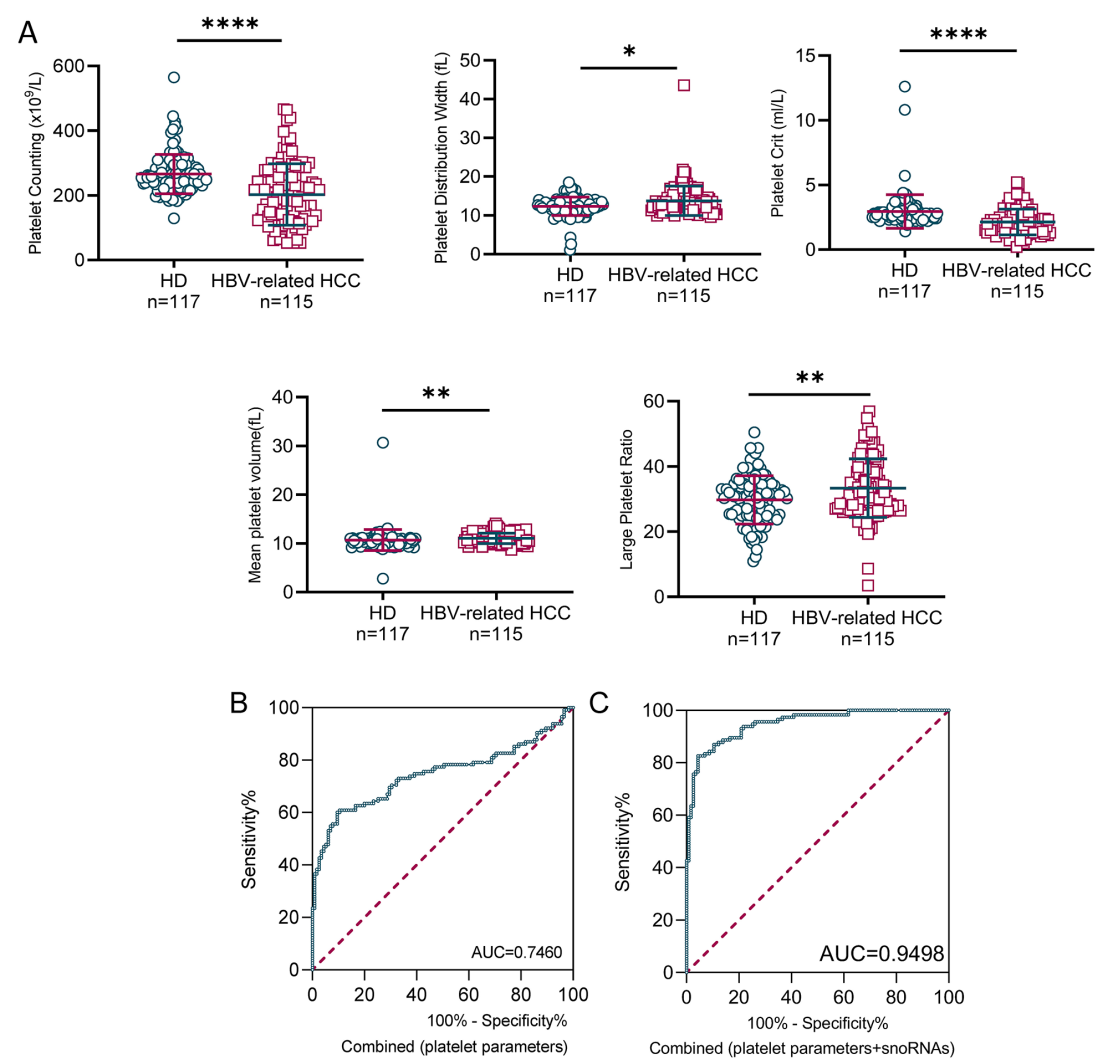
TEP snoRNAs combined with platelet parameters achieve favorable diagnostics efficiency for HBV-related HCC. **(A)** The platelet parameters, including platelet counting, platelet distribution width, platelet crit, mean platelet volume, and large platelet ratio, were different in HBV-related HCC patients compared with healthy controls. The ROC curve analysis of platelet parameters for HBV-related HCC patients **(B)**, and combined with all three snoRNAs **(C)** (AUC: area under curve; *p < 0.05, ***p < 0.001, ****p < 0.0001)

We further explored the effect of five platelet parameters on the diagnostic performance of the TEP snoRNAs for HBV-related HCC patients, including platelet number, mean platelet volume, platelet crit, large platelet ratio, and platelet distribution width. We also analyzed the correlation between snoRNA expression and platelet parameters in patients with HBV-associated HCC. However, no statistical difference was found between the levels of the three TEP snoRNAs tables and platelet parameters (Table 3). Nonetheless, as shown in Fig. 5A, the platelet parameters, including platelet count and platelet crit were downregulated, whereas the large platelet ratio, platelet distribution width, and mean platelet volume were upregulated in HBV-related HCC patients compared with healthy controls. Notably, these platelet parameters also had a considerable diagnostic efficacy, possessing an AUC of 0.7460 with a sensitivity of 60.87%, and a specificity of 89.57% when combined (Fig. 5B). More importantly, platelet parameters could enhance the diagnostic ability of TEP snoRNAs, elevating the AUC from 0.9047 to 0.948, with the sensitivity of 82.61% and specificity of 95.65% (Fig. 5C and Table S4).

**Table 3:** Correlation analysis of TEP snoRNAs expression levels and platelet parameters in patients with HBV-related HCC

	Samples	SNORD12B		SNORD14E		SNORA63	
		Mean ± SD	*P* value	Mean ± SD	*P* value	Mean ± SD	*P* value
**Platelet counting(< 183 × **10^9^**/L)**	56	11.71 ± 1.638	0.7077	3.738 ± 1.975	0.683	6.035 ± 2.124	0.8062
**Platelet counting (≥ 183 × 10** ^9^ **/L)**	59	11.81 ± 1.71		3.942 ± 2.744		5.921 ± 2.804	
**Not available**	2						
**Platelet distribution width(< 12.8 fL)**	55	11.76 ± 1.562	0.8792	3.898 ± 2.818	0.6625	5.77 ± 2.275	0.3952
**Platelet distribution (≥ 12.8 fL)**	60	11.76 ± 1.774		3.792 ± 1.946		6.166 ± 2.669	
**Not available**	2						
**Platelet crit(< 2 ml/L)**	57	11.79 ± 1.624	0.8749	3.813 ± 2.065	0.8052	6.059 ± 2.267	0.7261
**Platelet crit (≥ 2 ml/L)**	58	11.73 ± 1.725		3.872 ± 2.693		5.895 ± 2.702	
**Not available**	2						
**Mean platelet volume (< 10.8 fL)**	57	11.85 ± 1.599	0.385	3.927 ± 2.909	0.5407	6.181 ± 2.552	0.3847
**Mean platelet volume(≥ 10.8 fL)**	58	11.67 ± 1.744		3.761 ± 1.765		5.776 ± 2.425	
**Not available**	2						
**Large platelet ratio(< 32.1)**	57	11.73 ± 1.621	0.919	3.851 ± 2.937	0.7667	5.848 ± 2.398	0.585
**Large platelet ratio (≥ 32.1)**	58	11.79 ± 1.728		3.835 ± 1.723		6.103 ± 2.584	
**Not available**	2						

**Bold values indicate statistically significant*p*values** (*p*** < 0.05)**

## Discussion

HCC is one of the most frequently diagnosed primary liver cancers and is characterized by high morbidity and mortality [16]. Although numerous effective therapeutic methods, such as surgical resection, chemotherapy, liver transplantation, and immunotherapy, have been used, the prognosis of HCC remains poor mainly due to delayed diagnosis [17–20]. Recent studies have shown that TEPs can carry disease-related biomolecular dysfunctions (such as RNA, proteins, and metabolites) that overflow cells, suggesting a role in diagnosing disease [21]. In current study, we demonstrated TEP SNORD12B, SNORA63, and SNORD14E function as novel biomarkers for HBV-related HCC.

SNORD12B belongs to the family of C/D box snoRNAs. It can bind to the phosphatase PP-1α and facilitate its nuclear aggregation [22], as well as regulate the selective polyadenylation of ZBTB4 playing a vital role in regulating glucose and lipid metabolism, and the proliferation of GBM cells [23]; SNORA63, a member of the H/ACA box snoRNAs family, is located on chromosome 3 and is also known as U107 small-nucleus RNA and small-nucleus E3, but no comprehensive literature focuses on its expression and function; SNORD14E is a member of the C/D box snoRNAs family. High expression of SNORD14E may have negative effects on disease-free survival (DFS) and relapse-free survival (RFS) in patients with endometrial cancer (EC) [24]. In vitro studies have indicated that SNORD14E can promote the proliferation, migration, and invasion of EC cells while inhibiting apoptosis.

In the current study, we present several lines of evidence to validate TEP SNORD12B, SNORA63, and SNORD14E as potential biomarkers of HBV-related HCC. First, TEP SNORD12B and SNORD14E levels were visibly decreased in HBV-related HCC patients, while TEP SNORA63 was visibly increased. TEP SNORD12B and SNORD14E were significantly downregulated and SNORA63 was significantly upregulated in patients with early-stage HBV-related HCC compared to healthy volunteers, demonstrating a favorable diagnostic efficiency, especially in early diagnosis. Secondly, AFP is the most important and commonly used diagnostic indicator for HCC, but its diagnostic accuracy is limited since it has a high false-negative rate for detecting small and early-stage tumors. In addition, AFP may be elevated in some benign liver diseases, such as chronic hepatitis and cirrhosis without HCC [25]. Here, we demonstrated that TEP snoRNAs can also be used as a supplement to AFP for the diagnosis of HCC. Thirdly, snoRNAs in platelets mainly originate from megakaryocytes, and participate in the differentiation and maturation of megakaryocytes, as well as the generation of platelets. Besides, snoRNAs in anucleate platelets are responsible for alternative splicing and the change of biological function and parameters in platelets. Therefore, we believe that there is an underlying relationship between platelet parameters and snoRNAs. As expected, TEP snoRNAs combined with platelet parameters achieve favorable diagnostics efficiency for HBV-related HCC.

However, some points in the present study should be taken into consideration. First, U6 was selected as an endogenous reference gene. Platelet contains splicing proteins and snRNAs including U1, U2, U4, U5, and U6. U6 is transcribed by RNA pol III, but others by RNA pol II [26], thereby usually used as the endogenous control qPCR of miRNA [27], as well as of snoRNA [13, 28]. In our previous study, we had already detected 10 snRNAs expression including U1, U2, U4, U5, U6, U7, U8, U12, U4ATAC, and U6ATAC in the platelet [29]. U6 other than U1, U2, and U5 was very stable and indifferentiable in platelets from healthy individuals and cancer patients. Therefore, U6 was one of the most stable reference genes for the normalization of transcript level in platelets [13], including platelet derived snoRNA [14]. Secondly, a previous study gives the contribution of different risk factors for liver cancer. It was estimated that HBV contributed to about 59.3% of liver cancer in China, 9.7% can be attributed to excess body weight, 8.7% to HCV, and 0.56% to ever alcohol drinking [6]. In the current study, we had enrolled 150 HCC patients, in which HBV-related HCC was 117 (78%); the non-HBV-related was 33 (22%). HCC can be caused by alcohol, HCV infection, and NAFLD, however, there simply aren’t enough cases to analyze every one. To avoid complications related to the heterogeneity of HCC patients, we discussed studies of HBV-related HCC patients only.

In summary, the present study provides evidence that aberrant expression of SNORD12B, SNORA63, and SNORD14E in TEPs could serve as novel and non-invasive biomarkers for HBV-related HCC diagnosis.

### Electronic supplementary material

Below is the link to the electronic supplementary material.

Supplementary Material 1

Supplementary Material 2. Fig. S1: TEP SNORD12B, SNORD14E, and SNORA63 expression in non-HBV-related HCC. (A) Compared to healthy volunteers, individuals with non-HBV-related HCC had lower expression of TEP SNORD12B and TEP SNORD14E and higher expression of TEP SNORA63. (B) Expression of TEP SNORD12B and TEP SNORD14E in non-HBV-related HCC and HBV-related HCC. (*p < 0.05, ***p < 0.001, ****p < 0.0001)

Supplementary Material 3. Figure S2: The relationship between platelet counts and the expression of TEP SNORD12B, SNORD14E, and SNORA63 expression. (A)The expression of SNORD12B/SNORD14E/SNORA63 was irregulated with the platelet count.

## Data Availability

Not applicable.
