# Supplementary material for: TEP SNORD12B, SNORA63, and SNORD14E as novel biomarkers for hepatitis B virus-related hepatocellular carcinoma (HBV-related HCC)
Source: Cancer Cell Int. 2024 Jan 2;24:3. doi: 10.1186/s12935-023-03179-z (PMC10763353; doi:10.1186/s12935-023-03179-z)
Supplement: Supplementary file 1 — Supplementary Material 1 [file 12935_2023_3179_MOESM1_ESM.docx]

**Table S1 Comparison of the performance of TEP SNORD12B, SNORD14E, SNORA63 and their combination for the diagnosis of HBV-related HCC based on 117 HCC patients and 117 healthy donor cohorts**

|  | **SNORD12B** | **SNORD14E** | **SNORA63** | **Combined** |
| --- | --- | --- | --- | --- |
| **AUC** | 0.8313 | 0.6366 | 0.6739 | 0.9047 |
| **95%CI** | 0.7817 to 0.8808 | 0.5649 to 0.7082 | 0.6050 to 0.7428 | 0.8675 to 0.9420 |
| **Sensitivity%** | 70.94 | 47.86 | 61.54 | 81.2 |
| **Specificity%** | 76.07 | 81.2 | 70.09 | 88.03 |

**Table S2 Comparison of TEP SNORD12B, SNORD14E, and SNORA63 and their combined diagnostic performance based on an early HBV-related HCC cohort**

|  | **SNORD12B** | **SNORD14E** | **SNORA63** | **Combined** |
| --- | --- | --- | --- | --- |
| **AUC** | 0.8386 | 0.6709 | 0.6826 | 0.9267 |
| **95%CI** | 0.7758 to 0.9013 | 0.5857 to 0.7560 | 0.6010 to 0.7641 | 0.8879 to 0.9655 |
| **Sensitivity%** | 96.58 | 59.83 | 61.54 | 88.89 |
| **Specificity%** | 53.7 | 70.37 | 72.22 | 79.63 |

**Table S3 Comparison of diagnostic efficacy of AFP, TEP SNORD12B, SNORD14E, SNORA63 and their combination in 67 HCC patients and 67 healthy donors**

|  | **AFP** | **SNORD12B** | **SNORD12B+AFP** | **Combined** |
| --- | --- | --- | --- | --- |
| **AUC** | 0.7827 | 0.8162 | 0.8505 | 0.9401 |
| **95%CI** | 0.6975 to 0.8679 | 0.7472 to 0.8852 | 0.7872 to 0.9139 | 0.9032 to 0.9769 |
| **Sensitivity%** | 98.51 | 71.64 | 71.64 | 82.09 |
| **Specificity%** | 67.16 | 77.61 | 83.58 | 91.04 |

**Table S4 Comparison of the efficacy of platelet parameters TEP SNORD12B, SNORD14E, SNORA63 and their combination in the diagnosis of HBV-related HCC**

|  | **Platelet counting** | **Platelet distribution width** | **Platelet crit** | **Mean platelet volume** | **Large platelet ratio** | **Combined (platelet parameters)** | **Combined (platelet parameter+SNORNAs)** |
| --- | --- | --- | --- | --- | --- | --- | --- |
| **AUC** | 0.7218 | 0.5912 | 0.7401 | 0.5992 | 0.61 | 0.746 | 0.9498 |
| **95%CI** | 0.6526-0.7910 | 0.5181-0.6642 | 0.6718-0.8084 | 0.5264- 0.6719 | 0.5378-0.6823 | 0.6793-.8127 | 0.9241 to 0.9755 |
| **Sensitivity%** | 51.3 | 25.22 | 55.65 | 83.48 | 88.7 | 60.87 | 82.61 |
| **Specificity%** | 97.44 | 92.31 | 93.16 | 33.33 | 31.62 | 89.57 | 95.65 |
