# Supplementary figures and images for: TEP SNORD12B, SNORA63, and SNORD14E as novel biomarkers for hepatitis B virus-related hepatocellular carcinoma (HBV-related HCC)
Source: Cancer Cell Int. 2024 Jan 2;24:3. doi: 10.1186/s12935-023-03179-z (PMC10763353; doi:10.1186/s12935-023-03179-z)

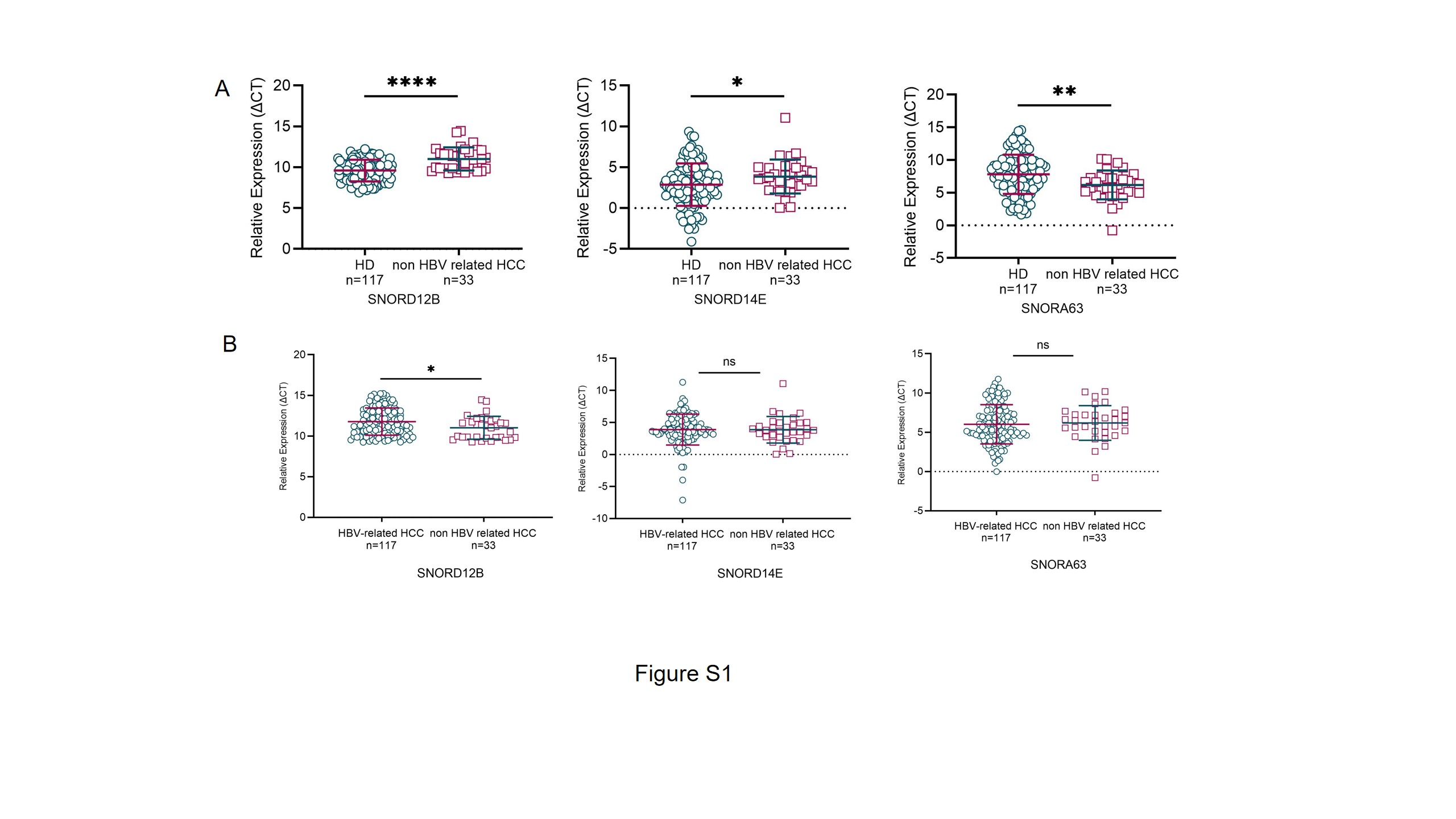

Supplement: Supplementary file 2 — Supplementary Material 2. Fig. S1: TEP SNORD12B, SNORD14E, and SNORA63 expression in non-HBV-related HCC. (A) Compared to healthy volunteers, individuals with non-HBV-related HCC had lower expression of TEP SNORD12B and TEP SNORD14E and higher expression of TEP SNORA63. (B) Expression of TEP SNORD12B and TEP SNORD14E in non-HBV-related HCC and HBV-related HCC. (*p < 0.05, ***p < 0.001, ****p < 0.0001) [file 12935_2023_3179_MOESM2_ESM.jpg]

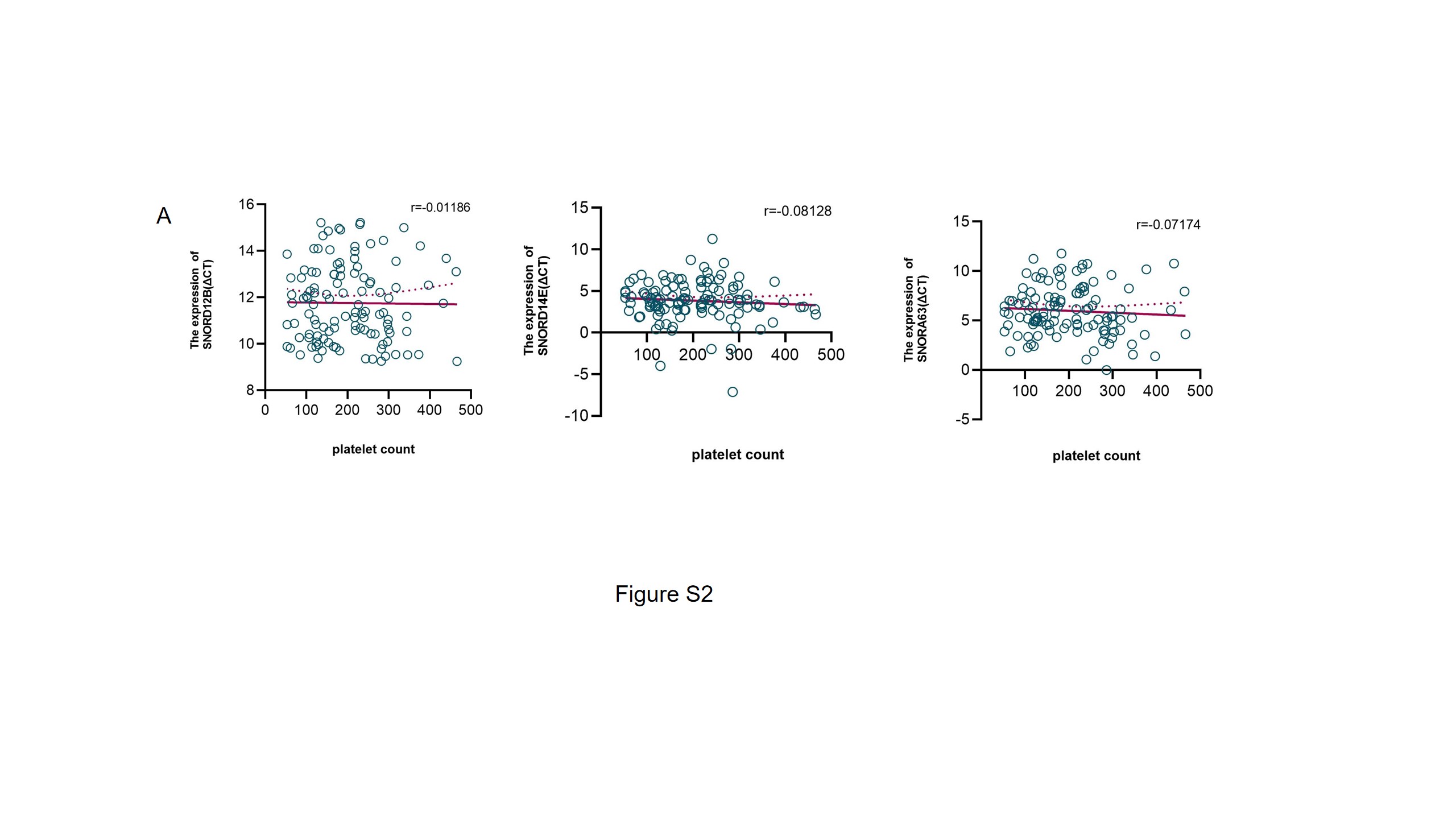

Supplement: Supplementary file 3 — Supplementary Material 3. Figure S2: The relationship between platelet counts and the expression of TEP SNORD12B, SNORD14E, and SNORA63 expression. (A)The expression of SNORD12B/SNORD14E/SNORA63 was irregulated with the platelet count. [file 12935_2023_3179_MOESM3_ESM.jpg]
